# Sodium Butyrate Modulates Mucosal Inflammation Injury Mediated by GPR41/43 in the Cecum of Goats Fed a High Concentration Diet

**DOI:** 10.3389/fphys.2019.01130

**Published:** 2019-08-30

**Authors:** Guangjun Chang, Nana Ma, Huanmin Zhang, Yan Wang, Jie Huang, Jing Liu, Hongyu Dai, Xiangzhen Shen

**Affiliations:** Department of Veterinary Clinical Science, College of Veterinary Medicine, Nanjing Agricultural University, Nanjing, China

**Keywords:** SCFAs, GPR41/43, inflammation, cecum, epigenetic mechanism

## Abstract

Emerging data indicate that excessive short chain fatty acids can mediate the downstream mitogen-activated protein kinase pathways by activating G-protein coupled receptor 41/43 (GPR41/43) to initiate the inflammatory response. The current study was conducted to investigate if a high concentrate (HC) supplemented with sodium butyrate can alleviate the inflammation and if an epigenetic mechanism is involved in regulating the expression of the key GPR41/43 genes in the cecum. Twelve lactating goats were randomly divided into two groups: the control group fed the HC diet and the treatment group fed the HC diet supplemented with sodium butyrate (HCB). Our results suggested that the supplementation of sodium butyrate significantly increased the pH value in the rumen and cecum, downregulated the expression of GPR41/43 and related inflammatory cytokines, upregulated the expression of tight junction proteins, and reduced the protein expression levels of GPR 41/43, ERK1/2, and p38. Moreover, the ratios of DNA methylation and chromatin compaction in the promoter region of the GPR41/43 genes were altered due to the addition of sodium butyrate. In brief, dietary addition of sodium butyrate can reduce the inflammatory injury to the cecal mucosa in lactating goats and can affect the expression of GPR41/43 via epigenetic modification.

## Graphical Abstract

Summary diagram of the antimicrobial resistance surveillance work developed by the UTAD Functional Genomics and Proteomics Unit.

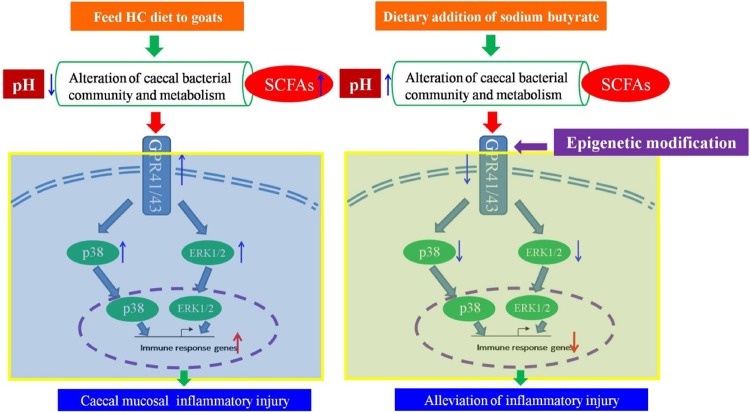

## Introduction

The cecum is an important fermentation organ in ruminants, including goats and cattle. Most of the chyme that has not been fermented completely in the rumen is fermented to produce final metabolites, such as short-chain fatty acids (SCFAs), in the cecum and other areas of the hindgut, and SCFAs are absorbed and utilized in the cecum (Faichney, 1968; Li et al., 2012). Some studies have reported that increasing the proportion of concentrate in the diet can improve the digestibility of diet, thereby produce a large amount of SCFAs in rumen and intestinal tract (Brink and Steele, 1985; Flachowsky and Schneider, 1992). SCFAs mainly include acetic acid, propionic acid, butyric acid, and other fatty acids with fewer than six carbon atoms in the carbon chain (Morrison and Preston, 2016). Excessive SCFAs in intestinal tract can lead to inflammatory bowel disease (IBD) by activating the GPR signaling pathway (Mihoshi and Chang, 2017). GPRs are transmembrane receptors that can be identified specifically by SCFAs, and GPRs, with a seven transmembrane α-helical structure, are coupled to a G protein; they are divided into five subfamilies according to their different ligands (Goldsmith and Dhanasekaran, 2007). Many studies have revealed that GPRs are drug targets that play an important role in various therapeutic processes and are the key molecules in certain signaling pathways involved in a series of physiological and pathological processes (Kimura, 2014).

A previous study has indicated that GPR41 mRNA is widely expressed in many organs and tissues, such as the pancreas, spleen and lymph nodes, and is especially prevalent in adipose tissue (Hong et al., 2005). GPR43 mRNA is detected in all tissues, and it is expressed more widely than GPR41 and at higher levels in immune cells, such as neutrophils, monocytes, peripheral blood mononuclear cells, and B lymphocytes (Nilsson et al., 2003; Nakajima et al., 2004); the expression levels of GPR41/43 were detected in gastrointestinal and endocrine cells (Karaki et al., 2006), suggesting that GPR41/43 might be involved in the occurrence and development of the immuno-inflammatory response. Many researchers have indicated that SCFAs activate GPR41 and GPR43, thus mediating mitogen-activated protein kinase (MAPK) cascade reactions with downstream inflammatory signaling pathways via different G protein subunits (Stoddart et al., 2008; Kim et al., 2013). GPR41/43 regulate MAPKs (ERK, JNK, p38 MAPK, etc.) by different mechanisms involving the α-, β-, and γ-subunits of the G protein (Goldsmith and Dhanasekaran, 2007). Other studies have shown that SCFAs activate GPR41/43, leading to MAPK signaling and producing most of the cytokines (IL-1β, TNF-α, IL-8, etc.) and chemokines (CCL5, CXCL10, etc.) in intestinal epithelial cells (Tarrerias et al., 2002; Kim et al., 2013). Previous study indicated that sodium butyrate improves immune function of organism and maintains homeostasis of intestinal environment (Peterson et al., 2008; Machiels et al., 2014). For example, it can attenuate and inhibit inflammation in IBD, such as ulcerative colitis (Vieira et al., 2012; Aguilar et al., 2014; Chang et al., 2018b). In addition, some studies have demonstrated that sodium butyrate modulates the expression of related epigenetic modification genes, such as EHMT2 (euchromatic histone-lysine-*N*-methyltransferase 2) (Candido et al., 1978), and HDAC11 (histone deacetylase-11) (Terova et al., 2016), indicating that sodium butyrate may be involved in DNA methylation and chromatin remodeling to regulate target gene expression via an epigenetic mechanism (Xu et al., 2015; Lee et al., 2017).

Previous studies have shown that feeding a high concentration (HC) diet enhances the expression of inflammatory cytokines and causes cecal inflammation in goats (Liu et al., 2014). In the present study, we hypothesized that sodium butyrate supplementation of the HC diet may alleviate inflammatory injury in the cecum and that the processes underlying this alleviation are involved in epigenetic modification via regulating the expression of the key genes GPR41/43.

## Materials and Methods

### Animals and Experimental Design

All experimental procedures were approved by the Institutional Animal Care and Use Committee of Nanjing Agricultural University. The sampling procedures were conducted strictly under the rules of “Guidelines on Ethical Treatment of Experimental Animals” (2006) No. 398 established by the Ministry of Science and Technology, China and the “Regulation regarding the Management and Treatment of Experimental Animals” (2008) No. 45 established by Jiangsu Provincial People’s Government. The HC diet formula is detailed in Supplementary Table S1. Twelve mid-lactation dairy goats (mean ± SEM body weight 34 ± 1.52 kg, milk yield 1.98 ± 0.56 kg/day) were hosted in individual cages at the Experimental Animal Centre (Nanjing Agricultural University, China). All goats were fed the HC diet for 4 weeks as an adaption period to achieve a similar metabolic status before initiation of the experiment and then randomly divided into two groups: a control group (HC, *n* = 6) fed the HC diet and a treatment group (HCB, *n* = 6) fed the HC diet supplemented with sodium butyrate (Dongying Degao Biotechnology, China). At the beginning of the second adaptation week, the goats were subjected the rumen fistula installation surgery to monitor dynamic changes in pH value in the rumen fluid. All goats were fed and milked at 8:30 a.m. and at 4:30 p.m. and were provided free access to fresh water; their body health was monitored by measuring body temperature and milk SCCs daily. After the adaption period, the formal experiment lasted for 8 weeks. During the formal experiment, the HC diet (2000 g DM/animal per day) was administered to each goat, the feeding program was described in Supplementary Table S2. The contents of net energy (5.83% DM) and crude protein (10% DM) in HC diet met or slightly exceeded the requirement for maintenance and lactation of dairy goats, according to the guidelines of NRC (Smith et al., 1969).

### Rumen Fluid, Cecal Tissues, and Contents

Rumen fluid was sampled before feeding (0 h) and at 2, 4, 6, and 8 h after feeding on the last three consecutive days of the 8^th^ week. All rumen fluid samples were filtered with medical gauze, and a small amount was collected to measure the pH value by using a pH meter (Sartorius, Germany). The remaining rumen liquid samples were kept at −20°C. After all goats were anesthetized and slaughtered, the cecum tissues were washed with saline and collected into frozen pipes (2 mL). After anesthesia, goat was slaughtered to obtain cecum tissue. And cecum tissue was washed three times with ice-cold 0.9% saline and then divided into two portions. The first portion was cut into approximately 1.0 cm × 0.5 cm pieces, and then these pieces were immediately frozen in liquid nitrogen for RNA expression determination. The second portion was cut into about 1.5 cm × 1.5 cm pieces, and mucosal tissues was separated from these pieces and then immediately transferred into liquid nitrogen for DNA extraction. Cecum contents were collected in 50-mL Eppendorf tubes for determination of the cecal pH value and the concentration of SCFAs (Liu et al., 2014). Fresh cecal contents were mixed completely with an equal volume of normal saline, and then the mixture was filtered by using gauze; the filtrate was saved for measuring the cecal content acidity. After diluting 10 g cecal contents with a crotonic acid/chloroform internal standard solution in 10 mL normal saline, the sample was acidified [w(Na_2_SO_4_)/v(50%H_2_SO_4_)/w(kieselguhr) = 30:1:20] and vortexed briefly; then, the supernatant collected. Crotonic acid served as the internal standard to determine the SCFA concentration using the gas chromatography method. The SCFA concentration for all samples was calculated based on the standard curve established by the respective reference standard materials. The method was performed as described previously with some modifications (Hamada et al., 1968). In brief, the current protocol of SCFAs used an FFAP 123-3233 30 m × 0.32 mm × 0.5 μm capillary column (Agilent Technologies, Santa Clara, CA, United States) in an Agilent 7890A system (Agilent Technologies).

### RNA Extraction and Real-Time Polymerase Chain Reaction

Tissue powder (100 mg) was weighed, and total RNA was extracted from the tissue using the RNAiso Plus Kit (TaKaRa, Japan) according to the manufacturer’s instructions. The concentration of total RNA was determined using a NanoDrop One (Thermo, Waltham, MA, United States), and the quality of the RNA was assessed by agarose gel electrophoresis. Then, cDNA synthesis was performed immediately using a reverse transcription reaction via the 5× PrimerScript^®^ RT Master Mix Kit in a 20-μL volume according to the manufacturer’s instructions. The primers for RT-qPCR were synthesized by Generay Biotech, Co. (Nanjing, China), and RT-qPCR was performed according to the SYBR Premix EX Taq^TM^ Kit (TaKaRa, Japan) instructions using an ABI7300 PCR apparatus. We used the absolute quantification analysis to assess the expression of related genes, as presented in a previous study (Chang et al., 2015). The sequences of primers for amplification are listed in Supplementary Table S3.

### Protein Preparation and Western Blot Analysis

Tissue powder (100 mg) was weighed, and total protein was extracted from the tissues using RIPA lysis buffer (Biosharp, Hefei, China). The concentration of total protein was assessed using the BCA Protein Assay kit (Pierce, Rockford, IL, United States). All protein samples were uniformly diluted to a concentration of 4 μg/μL and boiled in Laemmli sample buffer for 15% SDS-PAGE gel electrophoresis to separate the target protein. The separation of the predyed protein marker (Thermo, Waltham, MA, United States) was used to determine the target protein gel band, and the protein was transferred onto a nitrocellulose membrane. All membranes were blocked with 7% skim milk dissolved in Tris-buffered saline with Tween (TBST) for 2 h at room temperature. The membranes were then carefully washed with TBST and incubated with corresponding primary antibodies at 4°C overnight or for 12 h. Subsequently, the primary antibodies were recovered and kept at 4°C, and the membranes were washed individually with TBST 6 times (10 min each time) prior to incubation with the appropriate secondary antibodies at room temperature for 2 h. Finally, the membranes were washed individually with TBST 6 times (10 min each time) and then subjected to an enhanced chemiluminescence detecting kit (Biosharp) to expose the membranes using an imaging system (Bio-Rad, Hercules, CA, United States). The chemiluminescence image signals from the membranes were recorded and analyzed with Image Lab software provided with the system. In addition, the primary and secondary antibodies were diluted in TBST. The following antibodies were used: goat-anti GPR41 (1:500, BS5750, Bioworld), goat anti-GPR43 (1:200, sc-293202, Santa Cruz Biotechnology), mouse anti-p38 MAPK (1:200, AM065-1, Beyotime, Shanghai, China), rabbit anti-ERK1/2 (1:500, AF1051, Beyotime), mouse anti-β-tubulin (1:200, Kang Chen Biotech, China), and mouse anti-GAPDH (1:200, AG019-1, Beyotime) primary antibodies; donkey anti-goat (1:5000, Santa Cruz Biotechnology, Santa Cruz, CA, United States), goat anti-rabbit (1:5000, A0208, Beyotime) and goat anti-mouse (1:5000, A0216, Beyotime) horseradish peroxidase (HRP)-conjugated secondary antibodies.

### Genomic DNA Preparation for DNA Methylation of GPR41/43

Cecum tissue powder (100 mg) was weighed, and genomic DNA was extracted from the tissues using 3 ml proteinase K working buffer mixed with proteinase K (20 mg/ml), dithiothreitol (DTT, 1 mol/L) and SDS (20%) and incubated in a 56°C waterbath overnight. Then, the genomic DNA was obtained by using the standard isopropanol-precipitating method. Genomic DNA was completely diluted in double-distilled water (dd H_2_O). The DNA concentration was measured using a NanoDrop One (Thermo, United States), and then all the DNA samples were diluted uniformly in 200 ng/μL. DNA methylation was assessed by using a double enzyme (*Msp*I/*Hpa*II) digestion method; these two enzymes can identify the CpG site. Genomic DNA was digested with *Msp*I, *Hpa*II and control treatment (dd H_2_O) at 37°C for 2 h. After digestion, the genomic DNA was purified and diluted, and the concentration determined. The same amount (200 ng) of purified DNA was sampled for quantification, and amplification by RT-qPCR was used to determine the relative copy number and the rate of DNA methylation (ratio = $\frac{copiesofcontrol-copiesofHpaII}{copiesofcontrol-copiesofMspI}$) in each sample. The DNA sequences of GPR41/43 were analyzed using the NCBI online database and seqbuilder software. The primers for RT-qPCR were designed based on the GPR41/43 core promoter region and synthesized by Generay Biotech, Co. (Nanjing). The respective DNA copy numbers were calculated based on the dilution series (106 to 102 copies) of the respective cloned amplicons as external standards using the following sequences of primers: GPR41_CM (Forward primer: CTGTC TCTAC AGATC TGCCT AC, Reverse primer: CCGAA CATTA CCTGG TGTCC ATC, GenBank ID: NW_005100869) and GPR43_CM (Forward primer: TCACT TCACT TCACC ACGTG, Reverse primer: CCACT CGCCC AGAGA TCCTC AC, GenBank ID: NW_005100869).

### Chromatin Preparation and Chromatin Accessibility by Real-Time PCR (CHART-PCR)

Chromatin of cecum tissues was extracted by using the following steps: 100 mg tissue powder was weighed and added to 3 ml prechilled resuspension buffer I (RSB: 10 mM Tris (pH = 8.0); 3 mM MgCl_2_; 10 mM NaCl) containing 0.5% NP 40 diluted in dd H_2_O, which was followed by incubation on ice for 5 min. The proteinase inhibitor cocktail (PIC) was diluted at a ratio of 1:200, and the phenyl-methyl-sulfonyl-fluoride (PMSF, 1 mM) was prepared freely prior to use. After incubation, the liquid mixture was suspended and extracted manually with a pestle (Sigma-Kontes Glass Co-7ml A, United States). The fluid was filtered into a new precooled Eppendorf tube with sterilized glass wool, and the condition of the nuclei in the filtrate was examined under a microscope. The filtrate liquid was then centrifuged (1000 *g*, 10 min) at 4°C to precipitate the nuclei. The pellet was washed and mixed with RSB II (RSB added to 1 mM β-mercaptoethanol) and then centrifuged (3500 rpm, 10 min) at 4°C, producing a precipitate. Finally, the nuclear precipitate was diluted in RSB III (containing 50% glycerol) and stored at −20°C. Chromatin compaction analysis was performed with CHART-PCR. Nuclear DNA was digested with *Msp*I (mixed with PIC, PMSF in the enzyme digestion system) and the control treatment (dd H_2_O) for 30 min at 37°C in a waterbath, and then the system was added and mixed with an equal volume of 2× proteinase K working buffer (as previously mentioned) and incubated at 56°C for 2 h. After incubation, the nuclei were purified using the standard isopropanol-precipitating method. The DNA concentration was determined using a NanoDrop One. For all samples, 50 ng nuclear DNA was used for RT-qPCR, and the relative copy numbers of the respective nuclear DNA samples were calculated based on the quantitative standard curve for assessing the level of chromatin compaction.

### Statistical Analysis

All data in this study are shown as the “mean ± SEM.” Data of rumen pH values were analyzed as repeated measures using SAS software MIXED procedures (SAS version 9.4, SAS Institute, Inc.). The effects of diet, time and day were considered as fixed factor, and the effect of goat was considered as random factor. The effects of time and day was considered as repeated measure. The expression of related mRNAs and proteins were analyzed with the paired Student’s *t*-tests using IBM SPSS Statistics 20.0 (IBM, Inc., United States), and the correlation between the level of chromatin compaction and the expression of mRNA and between the level of chromatin compaction and the rate of DNA methylation were analyzed with Pearson’s test using IBM SPSS Statistics 20.0. *P* represents a significant difference; *P* < 0.050 is represented by “^∗^” and *P* < 0.010 by “^∗∗^” in all tables and figures.

## Results

### Rumen and Cecum pH

The rumen pH was higher in the HCB group than the HC group (*P* < 0.01, Table 1). Sodium butyrate addition significantly increased the pH value in the cecum of lactating goats in the HCB group compared with the HC group (*P* < 0.05).

**TABLE 1:** The pH value in the rumen and cecum of lactating goats.

**Item**	**HC**	**HCB**
Rumen pH value	5.62 ± 0.07	6.04 ± 0.21^∗∗^
Cecal pH value	6.06 ± 0.43	6.46 ± 0.48^∗^

*The data are displayed as the mean ± SEM, and significance between HC and HCB groups is indicated by ^∗^*P* < 0.050 or ^∗∗^*P* < 0.010.*

### The Concentration of SCFAs in the Cecum

**FIGURE 1:**
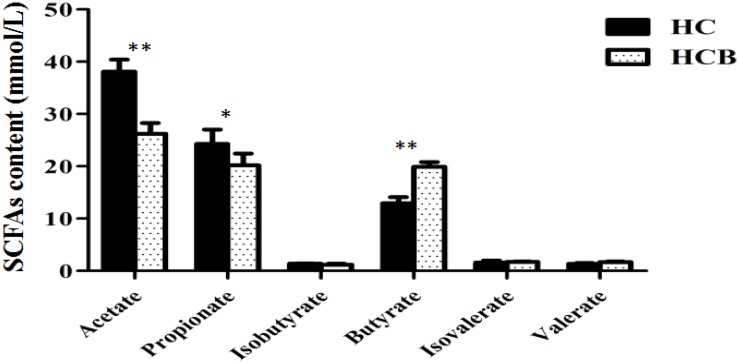
Effect of sodium butyrate on the content of SCFAs in the cecum of lactating goats. The SCFA levels are indicated on the ordinate axis (mean ± SEM). Significant differences between HC and HCB groups are represented by ^∗^*P* < 0.050 or ^∗∗^*P* < 0.010.

**FIGURE 2:**
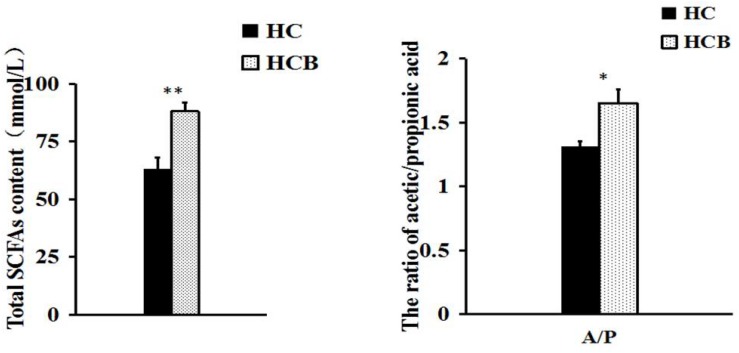
Effect of sodium butyrate on the content of total SCFAs and the ratio of acetic/propionic acid in the cecum of lactating goats. The data from both groups are indicated on the ordinate axis (mean ± SEM). Significant differences between HC and HCB groups are represented by ^∗^*P* < 0.050 or ^∗∗^*P* < 0.010.

Acetate (*P* < 0.01, Figure 1) and propionate (*P* < 0.05, Figure 1) contents were lower in the HCB group than the HC group. Sodium butyrate (*P* < 0.01, Figure 1) was elevated in the HCB group compared with the HC group. Other acids (*P* > 0.10, Figure 1) were not significantly different between the HC and HCB groups. The total SCFA content (*P* < 0.01, Figure 2) and the ratio of acetic/propionic acid (*P* < 0.01, Figure 2) was higher in the HCB group than the HC group.

### mRNA Expression of Inflammatory Response Genes in the Cecum

**FIGURE 3:**
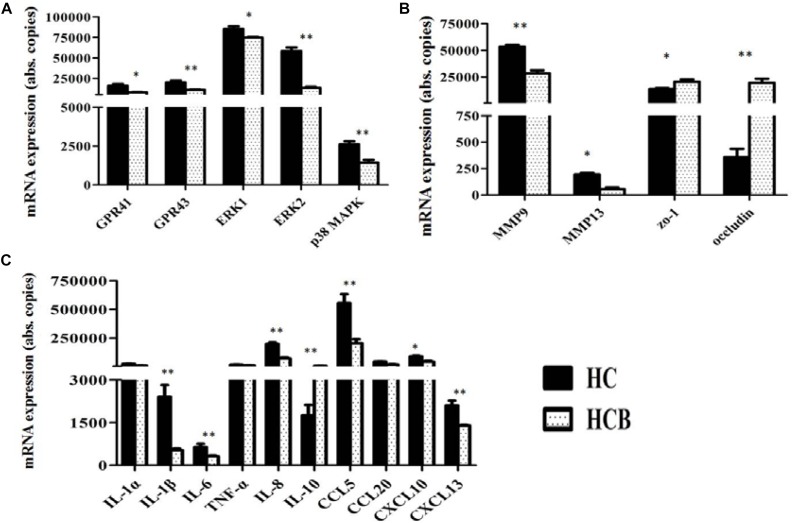
Effect of sodium butyrate on the expression of candidate genes in the cecal mucosal tissue of lactating goats. The levels of candidate gene expression are indicated on the ordinate axis (mean ± SEM). **(A)** Expression levels of GPR41/43 and transcription factors, **(B)** MMPs and TJPs, **(C)** and inflammatory cytokines and chemokines. Significant differences between HC and HCB groups are represented by ^∗^*P* < 0.050 or ^∗∗^*P* < 0.010.

Compared with the HC group, GPR41 (*P* < 0.05, Figure 3A) and GPR43 (*P* < 0.01, Figure 3A) mRNA expression levels were down-regulated in the HCB group, and the mRNA expression levels of ERK1 (*P* < 0.05, Figure 3), ERK2 (*P* < 0.01, Figure 3A), and p38 MAPK (*P* < 0.01, Figure 3A) (the genes associated with MAPKs pathway) were also down-regulated in the HCB group. Compared with the HC group, the mRNA expression levels of MMP9 (*P* < 0.01, Figure 3B) and MMP13 (*P* < 0.05, Figure 3B) were decreased in the HCB group, and those of zo-1 (*P* < 0.05, Figure 3B) and Occludin (*P* < 0.01, Figure 3B) were increased in the HCB group. The mRNA expression of the related inflammatory cytokines, IL-1β, IL-6, and IL-8, and chemokines, CCL5, CXCL13 (*P* < 0.01, Figure 3C) and CXCL10 (*P* < 0.05, Figure 3B), were significantly decreased, and the mRNA expression of IL-10 (*P* < 0.01, Figure 3C) was significantly increased in the HCB group. The expression levels of IL-1α, TNF-α, and CCL20 (*P* > 0.10, Figure 3C) did not differ between these two groups.

### Protein Expression of the GPR41/43 and MAPK Pathways

**FIGURE 4:**
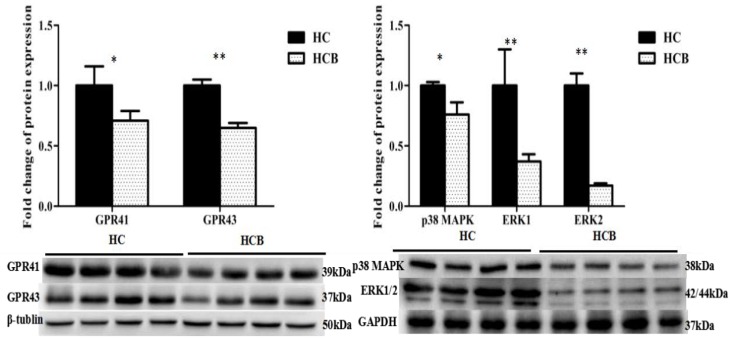
Effect of sodium butyrate on the protein expressions of GPR41/43 and MAPKs protein in the cecal mucosal tissue of lactating goats. Fold changes in protein expression are indicated on the ordinate axis (mean ± SEM), and significant differences (^∗^*P* < 0.050, ^∗∗^*P* < 0.010) are indicated. The bands (1–4) from left to right for each candidate protein represent the levels of the corresponding proteins in the cecal mucosal tissue of lactating goats in the HC group. The bands (5–8) from left to right for each candidate protein represent the levels of the corresponding proteins in the cecal mucosal tissue of lactating goats in the HCB group.

The protein expression levels of GPR41 (*P* < 0.05, Figure 4) and GPR43 (*P* < 0.01, Figure 4) were lower in the HCB group than the HC group. The protein expression levels of p38 MAPK (*P* < 0.05, Figure 4), ERK1 (*P* < 0.01, Figure 4), and ERK2 (*P* < 0.01, Figure 4) were lower in the HCB group than the HC group.

### The Correlation Between the Chromatin Compaction Level and mRNA Expression of GPR41/43

**FIGURE 5:**
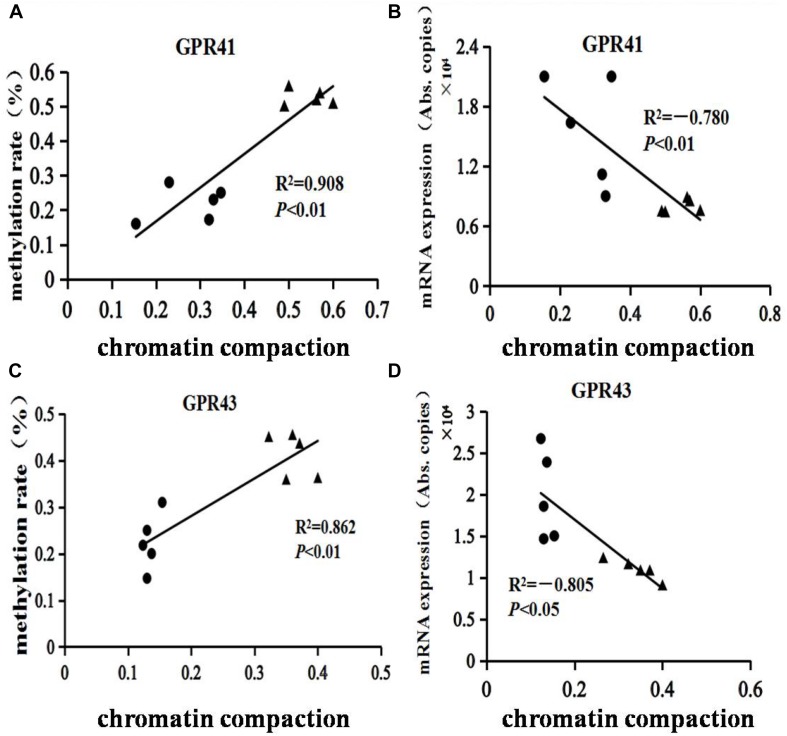
Assessment of chromatin compaction and DNA methylation of GPR41 and GPR43 in the cecal mucosal tissue of lactating goats. “
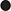
” represents the HC group, “▲” represents the HCB group. **(A,C)** Correlations between chromatin compaction and DNA methylation of GPR41 and GPR43. **(B,D)** Correlations between chromatin compaction and mRNA expression of GPR41 and GPR43. *R*^2^ is the correlation index, and *P* represents the significance of the correlation.

The structures of GPR41 and GPR43 promotors are drafted in Supplementary Figure S1. The GPR41 and GPR43 promoter methylation ratios are shown in Table 2. Compared with the HC group, the ratios of promoter methylation for GPR41 (*P* < 0.01) and GPR43 (*P* < 0.01) were increased in the HCB group. The correlation between the promoter methylation ratio for GPR41 and GPR43 in each sample and the corresponding chromatin compaction degree were analyzed, and the results for GPR41 (*R*^2^ = 0.91, *P* < 0.01, Figure 5A) and GPR43 (*R*^2^ = 0.86, *P* < 0.01, Figure 5C) revealed a positive correlation between the degree of chromatin compaction and the ratio of promoter methylation.

**TABLE 2:** Ratio of DNA methylation of the GPR41/43 promoter in the cecum.

**Gene (methylation, %)**	**HC**	**HCB**
GPR41	0.22 ± 0.02	0.62 ± 0.01^∗∗^
GPR43	0.24 ± 0.03	0.72 ± 0.02^∗∗^

*The data are displayed as the mean ± SEM, and significance between HC and HCB groups is indicated by ^∗∗^*P* < 0.010.*

The levels of chromatin compaction for GPR41 and GPR43 are shown in Table 3. Compared with the HC group, the levels of GPR41 (*P* < 0.01) and GPR43 (*P* < 0.01) were enhanced in the HCB group. The correlation between the mRNA expression of GPR41 and GPR43 in each sample and their corresponding chromatin compaction degree were analyzed, and the results for GPR41 (*R*^2^ = −0.78, *P* < 0.01, Figure 5B) and GPR43 (*R*^2^ = −0.81, *P* < 0.05, Figure 5D) revealed a negative correlation between the degree of chromatin compaction and mRNA expression.

**TABLE 3:** The level of chromatin compaction of GPR41/43 in the cecum.

**Gene (compaction, %)**	**HC**	**HCB**
GPR41	0.21 ± 0.06	0.49 ± 0.07^∗∗^
GPR43	0.17 ± 0.05	0.32 ± 0.03^∗∗^

*The data are displayed as the mean ± SEM, and significance between HC and HCB groups is indicated by ^∗∗^*P* < 0.010.*

## Discussion

Mounting data confirmed that long term feeding a HC diet to dairy goats or cows caused a series of consequences, such as the rumen pH depression, rumen flora disorder, gastrointestinal barrier injury, and multiple metabolic diseases, thereby resulting in huge economic losses in dairy industry (Plaizier et al., 2008; Dong et al., 2013; Chang et al., 2018a). Recent studies indicated that the addition of sodium butyrate in the HC diet attenuated rumen pH depression, altered the rumen fermentation state (Dai et al., 2017; Chang et al., 2018b). Our current data, consistent with previous study, showed that HCB diet feeding increased the rumen and cecum pH values and cecum SCFAs concentration compared with HC diet feeding, these results indicated that sodium butyrate addition improved gastrointestinal environment and microbial fermentation (Myers et al., 1967; Li et al., 2010, 2012). Furthermore, HC diet feeding significantly increased the concentration of acetate and propionate, especially acetate compared with HCB diet feeding. Previous study confirmed that the increases of acetate and propionate in cecum was one of the main reasons of cecal pH value depression (Tao et al., 2015).

Some studies demonstrated that administrating HC diet to dairy goats for more than 4 weeks leaded to subacute ruminal acidosis (SARA), which caused systemic inflammation (Chang et al., 2015; Bilal et al., 2016; Dai et al., 2017), including rumenitis, endometritis and liver injury and so on. Meanwhile, the inflammatory injury of cecal mucosa have also been verified by feeding high grain diet to goats for 7 weeks (Liu et al., 2014). A previous study showed that SCFAs activate GPR41/43 via eliciting the MAPK pathway to promote inflammatory responses in mouse intestinal epithelial cells (Kim et al., 2013); however, other researchers have found that SCFAs appear to have pro- and anti-inflammatory functions in macrophages and microglial cells (Eftimiadi et al., 1990; Niederman et al., 1997). In fact, acetate and some propionate are absorbed and transferred to the liver to synthesize glucose and other fatty acids, and butyrate and some propionate participate in cell replication and proliferation, water-electrolyte metabolism and leukocyte and neutrophil migration (Tvrzicka et al., 2011); further, SCFAs have also been shown to participate in leukocyte emigration and to modulate the secretion of certain cytokines (TNF-α, IL-6, IL-10, etc.) as well as their functions (Vinolo et al., 2011). Therefore, we detected the protein and mRNA expression levels of GPR41/43, MAPK and other related inflammatory genes, and the results demonstrated that the gene expression levels of GPR41, GPR43, ERK1/2, and p38 MAPK were lower in the HCB group than the HC group. Accordingly, compared with the HC group, the protein expression levels of GPR41, GPR43, ERK1/2 and p38 MAPK were decreased in the HCB group, and the expression levels of primary cytokine genes, IL-1β, IL-6, and IL-8, and chemokine genes, CCL5, CXCL13, and CXCL10, were lower in the HCB group than in the HC group. IL-10 expression was lower in the HCB group than in the HC group, but the expression of IL-1α, TNF-α, and CCL20 showed no difference between the HCB group and the HC group. These results suggested that sodium butyrate reduced the primary means of acetate and propionate production, downregulated the mRNA and protein levels of key receptors, GPR41/43, and signaling molecules, ERK1/2, p38 MAPK, and modulated the inflammatory cytokine and chemokine expression in the cecum of lactating goats, which is likely due to inhibitory effects of the high-concentrate diet supplemented with sodium butyrate on the inflammation elicited by HC feeding. In this study, we also assessed the expression of MMP9, MMP13, zo-1, and occludin associated with defense functions in the intestine. The mRNA expression levels of MMP9 and MMP13 were lower in the HCB group than the HC group; however, the mRNA expression levels of zo-1 and occludin were higher in the HCB group than the HC group. MMP9 and MMP13 belong to the MMP superfamily, are secreted by monocytes and neutrophils involved in inflammation and are related to cellular infiltration and migration. Their expression is also regulated via the p38 MAPK pathway (Wu et al., 2004), while zo-1 and occludin are known as tight junction proteins (TJPs), which function in maintaining epithelial barrier functions through signal transduction in epithelia and regulating the immune and inflammatory response. Numerous inflammatory cytokines, such as TNF-α, IFN-γ, and ILs, may modulate their expression (Youakim and Ahdieh, 1999). Research in IBD has demonstrated that sodium butyrate can protect the intestinal mucosa from damage and alleviate inflammation (Ahrens et al., 2008). The obtained results show that sodium butyrate actually plays an important role in protecting the intestinal mucosal functional integrity in the cecum.

We further investigated the DNA methylation rate and the level of chromatin compaction of GPR41/43 core promoter regions to evaluate the influence of sodium butyrate on the epigenetic mechanism. Epigenetic modification is influenced by many factors, including the diet, dietary supplements, and the environment, via DNA methylation, chromatin remodeling and histone acetylation, and thus regulates many physiological and pathological processes (Bayarsaihan, 2011). Some studies have verified that sodium butyrate is a dietary supplement that modulates the transcriptional regulation of some gene promoters via influencing epigenetic mechanisms in the intestine (Terova et al., 2016). We assessed the ratio of DNA methylation and the level of chromatin compaction of GPR41 and GPR43, respectively, and the results showed that the ratio of DNA methylation and the chromatin compaction level of the GPR41/43 promoter in the HCB group was higher than in the HC group, and the variations between the HCB and HC groups were in accordance with the GPR41/43 expression alterations. Thus, sodium butyrate is proposed to suppress the methylation and chromatin compaction and sequentially suppress GPR41/43 expression.

## Conclusion

The current study demonstrated that sodium butyrate, as a kind of dietary supplement, alleviates the inflammatory injury triggered by excessive SCFAs, mainly acetate and propionate, by activating GPR41/43 in the cecum of lactating goats fed a HC diet. Moreover, these alleviation processes are involved in epigenetic modification via regulating the expression of the key genes GPR41/43.

## Data Availability

All datasets generated for this study are included in the manuscript and/or the Supplementary Files.

## Ethics Statement

The animal study was reviewed and approved by the Institutional Animal Care and Use Committee of Nanjing Agricultural University.

## Author Contributions

GC conducted the experiment and drafted the manuscript. NM analyzed all the data. HZ, YW, JH, and JL collected the samples and performed the measurement of molecular parameters. XS conceived the idea, designed the experiments, and finalized the manuscript. All authors read and approved the final manuscript.

## Conflict of Interest Statement

The authors declare that the research was conducted in the absence of any commercial or financial relationships that could be construed as a potential conflict of interest.
