# Supplementary material for: Sodium Butyrate Modulates Mucosal Inflammation Injury Mediated by GPR41/43 in the Cecum of Goats Fed a High Concentration Diet
Source: Front Physiol. 2019 Aug 30;10:1130. doi: 10.3389/fphys.2019.01130 (PMC6729013; doi:10.3389/fphys.2019.01130)

**TABLE S1.** Dietary Ingredient and Nutrition Component

| （Ingredient） | | Percentage (%)of ingredients in different diets (dry matter) |
| --- | --- | --- |
| Chinese wildrye hay | | 26.70 |
| Alfalfa hay | | 13.30 |
| Corn | | 23.24 |
| Bran | | 20.77 |
| Soybean meal | | 13.66 |
| limestone | | 1.43 |
| Calcium phosphate dibasic | | 0.00 |
| Salt | | 0.40 |
| Premix ^a^ | | 0.50 |
| Forage : Concentrate(F:C) | | 4:6 |
| **Nutrient level, % of dry matter** | | |
| Net energy, MJ/kg | 5.83 | |
| CP, % | 10.00 | |
| NDF, % | 34.55 | |
| ADF, % | 20.35 | |
| NFC，% | 35.00 | |
| Ca，% | 0.90 | |
| P,% | 0.38 | |

^a^ Premix provided： 3000, 1250, and 40 IU kg^-1^ of diet of vitamin A, D and E, and 6.25, 62.5, 62.5, 50, 0.25, 0.125, 0.125 mg kg^-1^ of diet of Cu, Fe, Zn, Mn, I, Se, Co, respectively. CP: crude protein, NDF: neutral detergent fibre; ADF: acid detergent fibre, NFC: non-fibrous carbohydrate.

**TABLE S2.** Feeding Program of Lactating Goats

| Group | Concentrate（g/time） | Alfalfa（g/time） | Chinese Wild Rye（g/time） | Sodium Butyrate（g/time） |
| --- | --- | --- | --- | --- |
| HC | 600 | 80 | 320 | - |
| HCB | 600 | 80 | 320 | 10 |

**TABLE S3.** Primers and Length of Candidate Genes for RT-qPCR

| Genes | Forward primer | Reverse Primer | Length (bp) |
| --- | --- | --- | --- |
| GPR41 | CGGACTTGATCTGGAGGAGC | GTGAAGAAGAGGAACCTGGAG | 358 |
| GPR43 | TGGTAGCCTCAATGCCAGTC | TCTTTGCATCTGCGTCCCAA | 132 |
| IL-1α | GATGATGACCTGGAAGCCATTG | GCTGAGAATCCTCTTCTGATAC | 259 |
| IL-1β | CCGTGATGATGACCTGAGGAG | CAAGACAGGTATAGATTCTTGTC | 303 |
| IL-6 | CGAAGCTCTCATTAAGCACATC | CCAGGTATATCTGATACTCCAG | 241 |
| IL-8 | CTGAGAGTTATTGAGAGTGGGC | CAGTACTCAAGGCACTGAAGTAG | 259 |
| IL-10 | GTGATGCCACAGGCTGAGAAC | GAAGATGTCAAACTCACTCATGG | 213 |
| TNF-α | CAACAGGCCTCTGGTTCAGAC | GGACCTGCGAGTAGATGAGG | 209 |
| CCL5 | CTACACCAGCAGCAAGTGCT | CAAGCTGCTTAGGACAAGAG | 190 |
| CCL20 | GAAGCAGCAAGCAGCTTTGAC | GTTCCATTCCAGGGAGCATC | 244 |

**FIGURE S1.** The scheme shows map of GPR41/43 promoter core district including different transcriptional factor binding site (TFBS).


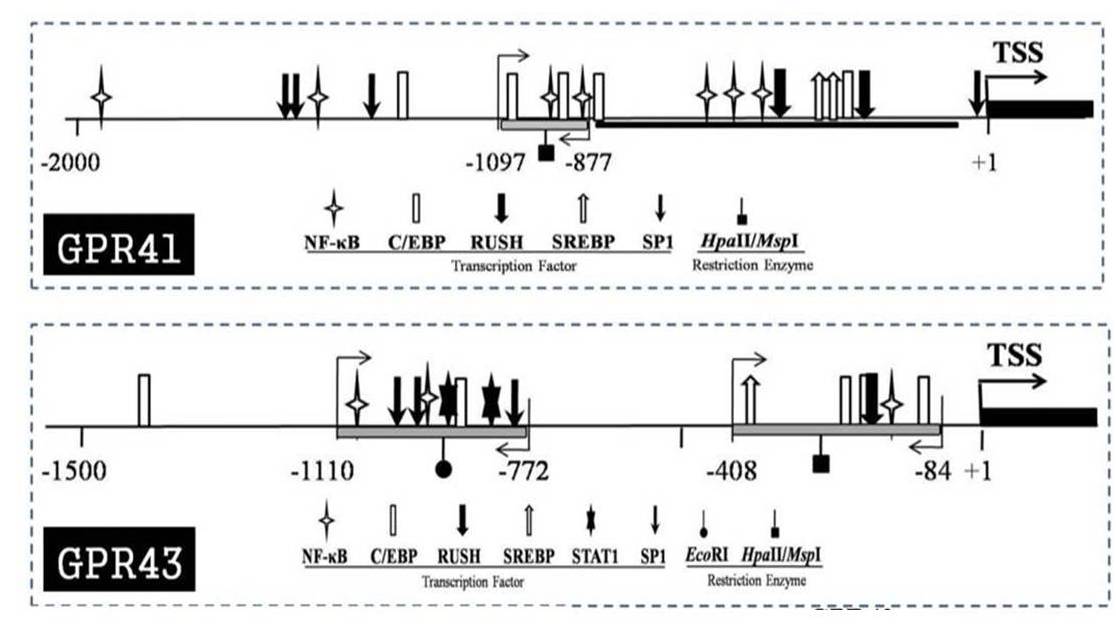

Supplement: Supplementary file 1 [file Table_1.DOCX]
